# Hospital Meetings

**Published:** 1896-01-04

**Authors:** 


### HOSPITAL MEETINGS.

### GREAT NORTHERN CENTRAL HOSPITAL.

### Special Meeting.

A largely-attended special meeting of the general council of the governors of the Great Northern Central Hospital was held on Monday, December 30th, in response to a requisition signed by certain governors calling in question the action of the Committee of Management in regard to the "commission and other emoluments paid to the late secretary" (Mr. W. T. Grant), "and the improper dealing with the same in the balance-sheets of ten annual reports, from 1885 to 1894."

Mr. Bax, the principal signatory to this requisition, was called upon by the Chairman (Mr. C. T. Murdoch, M.P.) to state his case. He complained that Mr. Grant received in salary and commission upon donations and legacies a yearly sum far exceeding his due; that in the year 1893 he drew altogether a sum of £1,011, on account of a large legacy falling in, and that on an average his yearly income was £750. He believed that Mr. Grant received, in addition to his salary of £300, 10 per cent. upon all new donations and 5 per cent. upon legacies, many of which, Mr. Bax contended, were in no way due to his exertions. He considered that Mr. Grant's salary alone was "a munificent remuneration" for his services. He was aware that it would be said that Mr. Grant rendered valuable service to the hospital, but he wished to call the attention of the governors to the fact that his predecessor (Mr. Baldwin) received a salary of only £150 and no commission. Mr. Bax went on to state that the governors had been grossly deceived on these matters for ten years, no account appearing in the yearly balance-sheet, the item "sundries" being really "secretary's perquisites." He asked the Committee of Management to explain these secret transactions, and concluded by moving a resolution to the effect that the meeting desired to express astonishment at the large sums paid to Mr. Grant, and at the secrecy observed in the matter.

Mr. Lines said he much regretted that he felt called upon to second this resolution, and he wished to propose that there should be a change made in the constitution of the Committee of Management.

Mr. Dodd said he understood that in the draft report of the year 1893 the auditors had made certain annotations in red ink against these payments, but that they were disregarded and no alteration was made in the report ultimately published. He would like to know whether these suggestions by the auditors came before the Committee of Management before the report was published.

After comments from one or two governors, Mr. Murdoch replied to the various charges brought forward. First, as to Mr. Grant's position. He did not see that Mr. Baldwin could be brought into the matter at all, because he was in no sense a permanent secretary; he held the position merely temporarily, for six months. Mr. Grant was elected at a very critical time in the history of the hospital. The Great Northern was a small hospital of thirty-two beds; an effort was to to be made to give that neighbourhood a great hospital to meet pressing requirements. It was of the utmost importance that a secretary should be appointed who was the best man possible for the post; and amongst those who devoted themselves to the matter, and took an enormous amount of trouble to find suitable candidates, was Mr. Burdett, whose name everyone knew stood high in matters of hospital management. The committee wanted a man who could not only administer a great hospital well, but who could also start it and set it going. Such a man was only available at a good salary. The question had been carefully considered, and it was a choice between a large inclusive salary or a small one with additional payment by results. Mr. Bax spoke of "huge amounts," but it would be seen that Mr. Grant's salary only averaged £750 per annum, including everything—not so much as was paid to their secretaries by other London hospitals. It was also to be borne in mind that Mr. Grant had been trained in a surveyor's office, and his experience had saved the hospital many expenses in connection with the building. Since his retirement, although their present secretary was most able, it had been necessary to appoint a steward at a salary of £150. All who were acquainted with hospital management knew that the Great Northern Central compared most favourably in expenses with other London hospitals. The expenditure per bed at the Metropolitan Hospital was £109, at Poplar £103, the French £78, North-West London £72, the German £68, Seamen's £64, and at the Great Northern Central £54 per bed. With regard to the other question, as to the auditors' report for 1893, in the usual course the balance-sheet, after being passed by the auditors, received the authority of the Finance Committee to be printed and published. On this particular occasion the usual form of balance-sheet was followed, and the auditors struck out the word "sundries," putting in "secretary's bonus, £519 5s. 9d." The Finance Committee approved that balance-sheet as passed by the auditors, and instructed that it should be printed: it was only after the reports had been circulated that it was discovered that the alteration had not stood. They took very good care that this mistake should not occur again, and the form of balance-sheet was forthwith altered to meet the wishes of the Hospital Sunday and Saturday Funds, stating most distinctly what commission, if any, is paid. With regard to the excessive amount of commission upon the legacy of the late Mr. Newman, in making the agreement with Mr. Grant the committee could not have foreseen the possibility of so large an amount accruing to the hospital; they took legal opinion, and they were bound to pay it. The arrangement with the present secretary was a very different one, and no commission whatever had been paid for the last two years. As to a re-constitution of the Committee of Management, all the members retired at the general meeting in February; they had done their best according to their lights, but if the governors wished them to give place to others they would cheerfully do so.

Dr. GLOVER then proposed as an amendment:—

That this meeting of governors, having heard the statement of Mr. Bax on the mode of remuneration of the late secretary, Mr. Grant, and the chairman's reply, expresses its satisfaction that the committee, of their own initiative, and some time prior to Mr. Bax's complaint, had already taken adequate steps to prevent any recurrence of the system *justly* complained of, and further records its confidence in the committee.

He thought Mr. Bax had done a service to his institution, and deserved the thanks of the governors. He had a great admiration himself for these committees, which did so much work, but thought they were sometimes the better for a little criticism. He felt it was now, however, a question whether Mr. Bax's resolution ought to be passed. The committee had made a bad contract with Mr. Grant in regard to the commission, but he was inclined to think that he did not, in fact, receive more than he was entitled to. He (Dr. Glover) spoke not only for himself, but as a member of the council of the Hospital Sunday Fund; and seeing that the Committee of Management had now discontinued the payment of commission, he thought the case would be met by passing the amendment he had proposed.

Mr. BURDETT said he particularly wished to second this amendment, as he should like his name to be associated with the committee so far as Mr. Grant's appointment was concerned. He had been a member of the committee which selected Mr. Grant. He reminded the governors that at the time of Mr. Grant's appointment the hospital had no funds to speak of, and that a great work lay before them for which an excellent administrator such as Mr. Grant was necessary. He thought the then circumstances justified payment by results, and that it was essential to the success of the hospital that Mr. Grant should be a partner with the committee, and depend for half his income upon his efforts. At first he was to receive 7½ per cent. upon all he got personally for the hospital. But afterwards this was thought too much, and the committee, two years or so after his appointment, made the grievous error of substituting for this limited commission of 7½ per cent. one of 2½ per cent. upon every thing. He felt strongly that the committee ought at once to have withdrawn the balance-sheet when they found that the auditors' correction had been omitted, though no doubt it would have required some courage on their part to take this step. A committee-man, however, without courage, was a man in the wrong place. He hoped that Mr. Bax, in view of its terms, would forego his original resolution in favour of Dr. Glover's amendment.

Mr. Bax offered to withdraw his amendment, but some of the Governors voted against this course.

After some further discussion Dr. Glover's amendment was adopted by a large majority.

Thereupon a motion was made to omit the word *justly*, which was resisted by the mover and seconder and by the independent governors present. It was, however, ultimately carried. The meeting terminated with a vote of thanks to the chairman.

